# Can head trauma trigger celiac disease? Nation-wide case–control study

**DOI:** 10.1186/1471-2377-13-105

**Published:** 2013-08-09

**Authors:** Jonas F Ludvigsson, Marios Hadjivassiliou

**Affiliations:** 1 Department of Pediatrics, Örebro University Hospital, Örebro, Sweden; 2 Clinical Epidemiology Unit, Department of Medicine, Karolinska Institutet, Stockholm, Sweden; 3 Division of Gastroenterology and Hepatology, Mayo Clinic College of Medicine, Rochester, USA; 4 Department of Neurology, Royal Hallamshire Hospital, Sheffield, UK

**Keywords:** Autoimmunity, Brain, Coeliac, Inflammation, Trauma

## Abstract

**Background:**

TG6, a brain expressed transglutaminase, is implicated in the neurological manifestations of celiac disease (CD). We hypothesized that earlier brain injury due to head trauma may be more common in patients with CD, potentially through trauma-induced TG6 leading to interaction with TG2.

**Methods:**

Through biopsy reports from all 28 pathology departments in Sweden we identified 29,096 individuals with CD (in this study defined as villous atrophy). We then examined the risk of earlier head trauma in CD compared to the risk in 144,522 controls matched for age, sex, county and calendar year. Odds ratios (ORs) were calculated using conditional logistic regression.

**Results:**

981 (3.4%) individuals with CD and 4,449 (3.1%) controls had a record of earlier head trauma. Individuals with head trauma were hence at a 1.10-fold increased risk of future CD (95% CI = 1.02-1.17). ORs were independent of sex or age at CD. The highest risk of future CD was seen during the first year after trauma. There was no association between severity of trauma and risk of developing CD.

**Conclusions:**

This study found a very small excess risk for future CD in individuals with an earlier head trauma.

## Background

Celiac disease (CD) occurs in about 1% of the population in Western Europe [1]. It is a multi-faceted disorder sometimes characterized by neurological manifestations such as gluten ataxia, [2] peripheral neuropathy [3,4], headache with white matter abnormalities, [5] and possibly also epilepsy [6,7]. Patients with gluten ataxia have positive serum antigliadin antibodies, but may not always have small intestinal enteropathy.

Our research has shown that a large proportion of individuals with gluten ataxia show increased levels of tissue transglutaminase 6 (TG6) [8,9]. Although TG6 originates in the brain, it has also been detected in the small intestinal mucosa, and can interact with TG2. It is capable of deamidating gliadin and it is gluten dependant [10].

CD is a lifelong small intestinal immune-mediated enteropathy characterised by inflammation and villous atrophy (VA). It occurs in about 1% of the Western population, and requires lifelong treatment with gluten-free diet. The overwhelming majority of individuals with CD are DQ2+ or DQ8+, but even if genetic studies have shown a high concordance rate of CD in monozygotic twins [11], genetic factors alone cannot explain the etiology of CD. In addition to the mandatory exposure to gluten, short breast-feeding [12,13], infections [13], caesarean section [14,15] and lack of smoking [16] have been implicated in the pathogenesis of CD. However much of earlier research on CD risk factors has actually shown contradicting data, and many of the positive findings have only been seen in small children with CD while the majority of individuals with CD are nowadays diagnosed in adulthood [17-19].

The aim of this study was to examine if individuals with head trauma leading to brain injury were at increased risk of developing CD. We hypothesized that head trauma and secondary cerebral and cerebellar insult may trigger autoimmunity against TG6 which in turn may lead, in genetically susceptible individuals, to the development of CD.

## Methods

We used data from small intestinal biopsy reports obtained from all 28 pathology departments in Sweden to identify individuals with CD. Biopsy data were then linked to inpatient and hospital-based outpatient data on head trauma through the unique Personal Identity Number assigned to all Swedish residents [20].

### Exposure - Head trauma

We defined head trauma (skull fractures and intracranial injury) as having a relevant international classification of disease (ICD) code in the Swedish Patient Register (*ICD7*: 800–4 and 851–55; *ICD8*: 800–4 and 850–54; *ICD9*: 800–4 and 850–54; and *ICD10*: S02 (minus S02.5) and S06). A recent validation found that the most common criteria for assigning an ICD code for head trauma in Sweden are loss of consciousness (76%) and posttraumatic amnesia (38%) [21].

The data source for head trauma, the Swedish Patient Register began in 1964 (then exclusively inpatient care) [22]. The coverage reached 75% of the Swedish population in the beginning of the 1980s, and the register became nationwide in 1987 [22]. Since 2001 the register also includes hospital-based outpatient visits.

### Outcome measure - Celiac disease

We collected data on VA (histopathology stage Marsh 3) [23] from computerized biopsy reports from all 28 Swedish pathology departments. The duodenal/jejunal biopsies had been performed between 1969 and 2008, but our data collection took place 2006–08. Searches for biopsy reports were carried out by local IT personnel who delivered data on personal identity number, morphology (for a list of relevant morphology codes according to the Swedish SnoMed system [19]), topography (duodenum or jejunum), and date of biopsy. Among 114 randomly selected individuals with VA undergoing patient chart validation, 108 (95%) had CD [19]. Biopsy reports were on average based on three tissue specimen [24] (and this should rule in about 95% of all CD [25]).

### Controls

Each individual with CD was matched with up to 5 controls for age, sex, county, and calendar year. Controls were sampled from individuals without a previous small intestine biopsy, and identified through the Swedish Total population register. Control matching was carried out by the government agency Statistics Sweden. After removal of duplicates and other data irregularities, our data-set was identical to that of our previous paper on mortality in CD [26]. The main analysis of this paper was hence based on 29,096 individuals with CD and 144,522 matched controls.

### Statistics

Conditional logistic regression was used to calculate odds ratios (ORs) for later CD in individuals with head trauma. We used a conditional approach; meaning that each individual with CD was first compared only to his/her matched controls within the same stratum. This eliminated the effect of age, sex, county and calendar year on our risk estimate. We then summarized stratum-specific data and calculated an overall OR for future CD.

A priori sub-analyses were carried out and included ORs according to sex, age and calendar period at CD diagnosis. We also examined the risk of CD according to time since head trauma (<1, 1–4.99, and ≥5 years).

In a sub-analysis we adjusted for country of birth (Nordic vs. not Nordic) and education according to four priori-defined categories [27]. Some 4% of individuals had no data on education and were fitted into a separate fifth category in the multivariate analysis.

In another analysis we examined head traumas occurring in adults (>19 years of age) and risk of future CD. We did so hypothesizing that non-nutritional risk factors may be especially important for adulthood CD.

We performed three sensitivity analyses. In two of these we examined the association between *severe* head trauma and CD. We defined severe head trauma as requiring either a) inpatient care in a neurosurgery department or b) ≥3 days of inpatient care. To increase the specificity of our exposure variable we also calculated the OR for CD in individuals with at least two prior records of head trauma (third analysis).

We used SPSS 18 (SPSS, Inc. Chicago, IL, USA) for all analyses. ORs with 95% confidence intervals that did not include one were regarded as statistically significant.

### Ethics

The current study was approved by the Ethics Review board of Stockholm, Sweden, which deemed that no individual informed consent was required since data were strictly register-based.

## Results

### Background data

The majority of the study participants were female (Table 1). The median age at diagnosis with CD was 30 years (range 0–95 years), and individuals had been diagnosed between 1969 and 2008 (median year: 1998). Some 96.7% of individuals with CD were born in the Nordic countries, compared to 94.3% of the controls. The median age at first head trauma in individuals with later CD was 18 years (range 0–92 years).

**Table 1:** Characteristics of study participants

	**Celiac disease**	**Matched controls**
Total, n	29,096	144,522
Females, n (%)	18,005 (61.9)	89,544 (62.0)
Males, n (%)	11,091 (38.1)	54,978 (38.0)
Age^a^ 0–19 years, n (%)	11,802 (40.6)	58,852 (40.7)
Age 20–39, n (%)	5,312 (18.3)	26,385 (18.3)
Age 40–59, n (%)	6,477 (22.3)	32,254 (22.3)
Age ≥60, n (%)	5,505 (18.9)	27,031 (18.7)
Calendar year^a^		
-1989, n (%)	4,105 (14.1)	20,378 (14.1)
1990-99, n (%)	12,059 (41.4)	59,874 (41.4)
2000-, n (%)	12,932 (44.4)	64,270 (44.5)
*Data on head trauma*		
Head trauma,^b^ n (%)	981 (3.4)	4,449 (3.1)
Age at first head trauma, years (median, range)	18; 0–92	19; 0-92

^a^At time of celiac disease diagnosis.

^b^Before diagnosis of celiac disease and matching date in controls.

### Main findings

Of 29,096 individuals with CD, 981 (3.4%) had a record of earlier head trauma compared to 4,449/144,522 (3.1%) controls. The OR for future CD in individuals with head trauma was 1.10 (95% CI = 1.02-1.17). Adjustment for education and country of birth did not affect the risk estimates (data not shown).

The highest risk for future CD was seen in the first year after head trauma (OR = 1.35; 95% CI = 1.10-1.66), with decreasing ORs thereafter (1–4.99 years: 1.21; 95% CI = 1.05-1.38). No association was seen between head trauma and future CD ≥5 years after the trauma (OR = 1.02; 95% CI = 0.94-1.11).

The OR for future CD was similar in males and females, but only in females did the association reach statistical significance (OR = 1.13) (Table 2). Head trauma in childhood increased the risk of having a later CD diagnosis in childhood (OR = 1.17; 95% CI = 1.03-1.32). Head trauma was actually associated with an increased risk of future CD in all age categories except for in the oldest age category where the association was neutral (Table 2). There was no difference in risk estimates according to calendar period (p for interaction: 0.099) (Table 2).

**Table 2:** Head trauma and risk of later Celiac disease

**Subgroup**	**Head traumas, N (%)**		**OR; 95% CI**	**P-value**	**P for interaction**
	Celiac disease	Controls			
Sex					0.323
Males	465 (4.2)	2,189 (4.0)	1.06; 0.95-1.17	0.292	
Females	516 (2.9)	2,260 (2.5)	1.13; 1.03-1.24	0.010	
Age^a^					0.103
<20 yrs	294 (2.5)	1,245 (2.1)	1.17; 1.03-1.32	0.013	
20-39 yrs	292 (5.5)	1,315 (5.0)	1.11; 0.98-1.26	0.112	
40-59 yrs	229 (3.5)	1,062 (3.3)	1.07; 0.93-1.24	0.330	
60+ yrs	166 (3.0)	827 (3.1)	0.98; 0.83-1.16	0.848	
Calendar period^a^					0.099
-1989	77 (1.9)	256 (1.3)	1.48; 1.15-1.90	0.002	
1990-1999	344 (2.9)	1,589 (2.7)	1.08; 0.96-1.21	0.215	
2000-2008	560 (4.3)	2,604 (4.1)	1.07; 0.98-1.17	0.153	

^a^At time of celiac disease diagnosis.

Restricting our analysis to head traumas occurring in adults we found a small excess risk of later CD (OR = 1.10; 95% CI = 1.03-1.18).

### Sensitivity analyses

We found no statistically significant associations between CD and head trauma, when we restricted our exposure to severe head trauma defined as either requiring inpatient care in a neurosurgery department (OR = 0.77; 95% CI = 0.51-1.16), or ≥3 days of any inpatient care (OR = 1.10; 95% CI = 0.96-1.27) (Table 3).

**Table 3:** Sub analyses: head trauma and risk of later celiac disease

**Subgroup**	**Head trauma requiring care in neurosurgery dept.**	**Head trauma with at least 3 days of inpatient care**	**At least 2 records of prior head trauma**
	OR; 95% CI	OR; 95% CI	OR; 95% CI
Overall	0.77; 0.51-1.16	1.10; 0.96-1.27	1.02; 0.86-1.20
Sex			
Males	0.84; 0.51-1.39	1.04; 0.85-1.26	0.77; 0.60-0.99
Females	0.65; 0.32-1.34	1.19; 0.96-1.46	1.38; 1.09-1.75
Age^a^			
<20 yrs	0.57; 0.14-2.40	1.18; 0.67-2.18	1.29; 0.93-1.79
20-39 yrs	0.25; 0.06-1.01	1.07; 0.79-1.44	0.84; 0.60-1.19
40-59 yrs	0.90; 0.41-1.98	1.17; 0.93-1.48	1.04; 0.74-1.46
60+ yrs	1.07; 0.61-1.90	1.04; 0.82-1.33	0.94; 0.66-1.35
Calendar period^a^			
-1989	0.84; 0.19-3.65	1.59; 1.06-2.37	1.33; 0.69-2.56
1990-1999	0.58; 0.27-1.25	1.01; 0.81-1.26	0.86; 0.62-1.20
2000-2008	0.89; 0.53-1.50	1.09; 0.89-1.35	1.06; 0.86-1.31

^a^At time of celiac disease diagnosis.

Having at least two records of head trauma was not associated with later CD (OR = 1.02; 95% CI = 0.86-1.20). Data from stratified analyses are given in Table 3.

## Discussion

In this nationwide case–control study we examined the association between head trauma and CD. Given the role of TG6 in the pathophysiology of gluten ataxia and its abundance in brain tissue, we hypothesized that head trauma resulting in cerebral and cerebellar insults may trigger autoimmunity against TG6 which in turn may lead in some genetically susceptible individuals, to the development of CD. This study found a slight but significantly increased risk of CD in individuals with previous head trauma.

TG6, TG2 (the autoantigen in CD) and TG3 (the autoantigen in dermatitis herpetiformis) share genetic, structural and enzymatic properties. It has been shown that patients with gluten ataxia have an immunological response primarily directed against TG6 even in the absence of enteropathy [8]. The interplay between these 3 types of transglutaminases during disease state is not yet known. What is known is that not all patients with CD are positive for TG3 or TG6 and not all patients with gluten ataxia are positive for TG2 or TG3. As these 3 transglutaminases have a 65% homology, are gluten dependants and are capable of deamidating gluten it is plausible that the development of autoimmunity to one may lead to the development of autoimmunity to the others perhaps through a process of epitope spreading. In gluten ataxia, there is evidence of IgA deposits against TG6 in the cerebellum [8]. TG6 autoantibodies have been thought to be a more specific marker for the neurological manifestations (in particular gluten ataxia), with the median TG6 antibody concentration being significantly higher than TG2 antibody in such patients.

During brain injury self epitopes (e.g. TG6), which are normally shielded from the systemic immune system, may become exposed to adaptive immunity. This may result in the immune system reacting to self-antigens in the central nervous system ultimately leading to autoimmunity. One study has demonstrated the presence of intrathecal antibodies against transglutaminases in the cerebrospinal fluid of patients with neurological dysfunction [28]. After brain injury, brain specific antigens can be measured in the serum implying that such antigens can gain access into the systemic circulation and are not restricted by the blood–brain barrier [29].

We are not aware of any earlier study that has examined if head trauma could trigger CD. In CD diagnosed in early childhood, nutritional factors are likely to play an important role in the CD pathogenesis, while less is known with regards to adulthood CD. Risk factors may exert different degrees of influence depending on the age of the individual. Due to the large statistical power of our study we were able to perform age-stratified analyses, but also specifically analyze the impact of head trauma in adulthood. Odds ratios were similar in all these analyses except for a lack of association among individuals aged ≥60 years at CD diagnosis. The lack of association between head trauma and CD in this age stratum could be due to limitations of the Swedish Patient Register. Although this register has nationwide coverage since 1987, and began in 1964 it will not have covered the childhood or adolescence (when some will have had head trauma) of many of those individuals diagnosed with CD ≥ 60 years. This is also obvious from Table 2 where the proportion of individuals with a record of prior head trauma in this age-group is lower than among younger adults. The median age at first head trauma was 18 years.

Although the association between head trauma and later CD was only statistically significant in women, we found no statistical heterogeneity between men and women. While ORs were not statistically significantly different between calendar periods there was a trend towards a higher OR before 1990. One explanation for this could be the lower awareness of CD in the 1970s and 1980s, allowing surveillance bias after head trauma to play a greater role for the detection of CD in these years.

We ascertained CD through biopsy data. This informed us about the exact date of CD diagnosis, but we have no data on the exact date of CD *onset*. Many individuals go undiagnosed for several years [30], and such misclassification may have affected our ORs. We did not screen study participants for CD, and can therefore not confirm or reject an association between head trauma and positive serology (including positive IgA gliadin antibodies) in individuals who did not contact health care. However the threshold for attending health care is low in Sweden since virtually all health care is publicly funded and virtually free of charge. However, a large proportion of individuals with CD remain undiagnosed [31], and we cannot rule out that the association with head trauma is restricted to clinically manifest CD.

In a Swedish setting VA is seldom due to other causes than CD [19]. When we manually examined more than 1500 biopsy reports with VA or inflammation, the most common non-celiac diagnosis mentioned in the biopsy reports was IBD constituting only 0.3% of the records. The most common symptoms in a subset of randomly selected individuals with CD were diarrhea (36%) and anemia (35%) [19] Biopsy reports with VA are also likely to have a high sensitivity for diagnosed CD since 96-100% of all gastroenterologists and pediatricians in Sweden perform a biopsy before CD diagnosis [19].

We used ICD codes to identify head trauma. Bellner *et al.* have shown that 91% of Swedish hospitals managing head injuries use the ICD-10 code “SO6” to code head trauma [21]; and we used the same ICD-9 codes as in a previous Swedish study on head trauma [32].

In order to increase the specificity of our exposure variable, we carried out several sensitivity analyses. These analyses showed inconsistent results with one showing a negative association, a second a positive association, and a third a neutral association between head trauma and CD. The 95% CIs of the three analyses did however overlap and it should be noted that head trauma requiring health care in a neurosurgery department, indicating severe head trauma did not show an association with future CD (OR = 0.77). This observation, however does not necessarily argue against our hypothesis because the severity of the insult to the brain may not correlate with the likelihood of the development of autoimmunity to TG6. Furthermore patients with head injury that are admitted to neurosurgical units are very likely to receive steroids as a means of reducing swelling secondary to the head injury. Steroids are known to interfere with the immune response.

Another explanation for the small increased risk of CD in our main analysis is surveillance bias. The highest ORs were seen in the first year after head trauma, and after that ORs went down towards 1. Head trauma can have long-term consequences including posttraumatic headache, necessitating regular health care contact where a CD may have been discovered only because the patient met with a physician for another reason (head trauma). However we cannot altogether rule out that the 10% excess risk of CD seen in this study is true, since it is consistent with our a-priori hypothesis. The observation that the highest ORs were seen in the first year after head trauma would be consistent with the timescale of an autoimmune response triggered by the monophasic event of head trauma without any chronic ongoing insult to the brain, but may also be due to increased testing for a number of diagnoses (including CD) in patients with brain trauma necessitating hospital contact.

Finally, our earlier research has shown that CD is associated with a number of other immune-mediated diseases [33-35] and such comorbidity, if linked to prior head trauma, may also have influenced our risk estimates.

## Conclusions

In conclusion, we found a small increased risk of CD in individuals with earlier head trauma. This excess risk may be due to surveillance bias or due to an autoimmune response against transglutaminases triggered by the brain injury.

## Details of ethics approval

This project (2006/633-31/4) was approved by the Research Ethics Committee of the Karolinska Institute, Sweden on June 14, 2006.

## Abbreviations

CD: Celiac disease; CI: Confidence interval; HR: Hazard ratio; VA: Villous atrophy.

## Competing interests

The authors declare that they have no conflict of interest.

## Authors’ contributions

ICMJE criteria for authorship read and met: JFL, MH. Agree with the manuscript’s results and conclusions: JFL, MH. Designed the experiments/the study: JFL, MH. Collected data: JFL. Analyzed the data: JFL. Wrote the first draft of the paper: JFL. Contributed to study design, interpretation of data and writing: MH. Interpretation of data; approved the final version of the manuscript: JFL, MH. Responsible for data integrity: JFL. Obtained funding: JFL. All authors read and approved the final manuscript.

## Pre-publication history

The pre-publication history for this paper can be accessed here:

http://www.biomedcentral.com/1471-2377/13/105/prepub
